# Supplementary material for: Equity-Informative Economic Evaluations of Vaccines: A Systematic Literature Review
Source: Vaccines (Basel). 2023 Mar 9;11(3):622. doi: 10.3390/vaccines11030622 (PMC10057152; doi:10.3390/vaccines11030622)
Supplement: Supplementary file 1 [file vaccines-11-00622-s001.zip › vaccines-2244550-supplementary.pdf]

Equity-informative economic evaluations of vaccines: a systematic literature review

SUPPLEMENTARY MATERIAL

Table S1. PRISMA 2020 Checklist

Section and Topic	Item #	Checklist item	Location where item is reported
**TITLE**			
Title	1	Identify the report as a systematic review.	Title
**ABSTRACT**			
Abstract	2	See the PRISMA 2020 for Abstracts checklist.	Abstract
**INTRODUCTION**			
Rationale	3	Describe the rationale for the review in the context of existing knowledge.	Introduction
Objectives	4	Provide an explicit statement of the objective(s) or question(s) the review addresses.	Introduction
**METHODS**			
Eligibility criteria	5	Specify the inclusion and exclusion criteria for the review and how studies were grouped for the syntheses.	Materials and Methods; Search strategy and Eligibility criteria
Information sources	6	Specify all databases, registers, websites, organisations, reference lists and other sources searched or consulted to identify studies. Specify the date when each source was last searched or consulted.	Materials and Methods; Search strategy and Eligibility criteria
Search strategy	7	Present the full search strategies for all databases, registers and websites, including any filters and limits used.	SUPPLEMENTARY MATERIAL Table S2
Selection process	8	Specify the methods used to decide whether a study met the inclusion criteria of the review, including how many reviewers screened each record and each report retrieved, whether they worked independently, and if applicable, details of automation tools used in the process.	Materials and Methods; Search strategy and Eligibility criteria
Data collection	9	Specify the methods used to collect data from reports, including how many reviewers collected data from each report, whether they worked	Materials and Methods;

Section and Topic	Item #	Checklist item	Location where item is reported
process		independently, any processes for obtaining or confirming data from study investigators, and if applicable, details of automation tools used in the process.	Data extraction
Data items	10a	List and define all outcomes for which data were sought. Specify whether all results that were compatible with each outcome domain in each study were sought (e.g. for all measures, time points, analyses), and if not, the methods used to decide which results to collect.	Materials and Methods; Data extraction
	10b	List and define all other variables for which data were sought (e.g. participant and intervention characteristics, funding sources). Describe any assumptions made about any missing or unclear information.	Not Applicable
Study risk of bias assessment	11	Specify the methods used to assess risk of bias in the included studies, including details of the tool(s) used, how many reviewers assessed each study and whether they worked independently, and if applicable, details of automation tools used in the process.	Materials and Methods; Quality assessment
Effect measures	12	Specify for each outcome the effect measure(s) (e.g. risk ratio, mean difference) used in the synthesis or presentation of results.	Not Applicable
Synthesis methods	13a	Describe the processes used to decide which studies were eligible for each synthesis (e.g. tabulating the study intervention characteristics and comparing against the planned groups for each synthesis (item #5)).	Materials and Methods; Data synthesis
	13b	Describe any methods required to prepare the data for presentation or synthesis, such as handling of missing summary statistics, or data conversions.	Not Applicable
	13c	Describe any methods used to tabulate or visually display results of individual studies and syntheses.	Materials and methods; Data synthesis
	13d	Describe any methods used to synthesize results and provide a rationale for the choice(s). If meta-analysis was performed, describe the model(s), method(s) to identify the presence and extent of statistical heterogeneity, and software package(s) used.	Materials and Methods; Data synthesis
	13e	Describe any methods used to explore possible causes of heterogeneity among study results (e.g. subgroup analysis, meta-regression).	Not Applicable
	13f	Describe any sensitivity analyses conducted to assess robustness of the synthesized results.	Not Applicable
Reporting bias assessment	14	Describe any methods used to assess risk of bias due to missing results in a synthesis (arising from reporting biases).	Not Applicable
Certainty assessment	15	Describe any methods used to assess certainty (or confidence) in the body of evidence for an outcome.	Not Applicable
**RESULTS**			
Study selection	16a	Describe the results of the search and selection process, from the number of records identified in the search to the number of studies included in the review, ideally using a flow diagram.	Results, Figure 1
	16b	Cite studies that might appear to meet the inclusion criteria, but which were excluded, and explain why they were excluded.	SUPPLEMENTARY

Section and Topic	Item #	Checklist item	Location where item is reported
			MATERIAL Table S3
Study characteristics	17	Cite each included study and present its characteristics.	Results, Tables 1 and 2, SUPPLEMENTARY MATERIAL Table S4
Risk of bias in studies	18	Present assessments of risk of bias for each included study.	Results; Quality assessment, Table 3
Results of individual studies	19	For all outcomes, present, for each study: (a) summary statistics for each group (where appropriate) and (b) an effect estimate and its precision (e.g. confidence/credible interval), ideally using structured tables or plots.	SUPPLEMENTARY MATERIAL Table S5
Results of syntheses	20a	For each synthesis, briefly summarise the characteristics and risk of bias among contributing studies.	Results, Table 2
	20b	Present results of all statistical syntheses conducted. If meta-analysis was done, present for each the summary estimate and its precision (e.g. confidence/credible interval) and measures of statistical heterogeneity. If comparing groups, describe the direction of the effect.	Not Applicable
	20c	Present results of all investigations of possible causes of heterogeneity among study results.	Not Applicable
	20d	Present results of all sensitivity analyses conducted to assess the robustness of the synthesized results.	Not Applicable
Reporting biases	21	Present assessments of risk of bias due to missing results (arising from reporting biases) for each synthesis assessed.	Not Applicable
Certainty of evidence	22	Present assessments of certainty (or confidence) in the body of evidence for each outcome assessed.	Not Applicable
**DISCUSSION**			
Discussion	23a	Provide a general interpretation of the results in the context of other evidence.	Discussion
	23b	Discuss any limitations of the evidence included in the review.	Discussion; Limitation
	23c	Discuss any limitations of the review processes used.	Discussion; Limitation
	23d	Discuss implications of the results for practice, policy, and future research.	Discussion; Limitation
**OTHER INFORMATION**			
Registration and protocol	24a	Provide registration information for the review, including register name and registration number, or state that the review was not registered.	Materials and Methods
	24b	Indicate where the review protocol can be accessed, or state that a protocol was not prepared.	Materials and Methods

Section and Topic	Item #	Checklist item	Location where item is reported
	24c	Describe and explain any amendments to information provided at registration or in the protocol.	Not Applicable
Support	25	Describe sources of financial or non-financial support for the review, and the role of the funders or sponsors in the review.	Funding
Competing interests	26	Declare any competing interests of review authors.	Conflict of interest
Availability of data, code and other materials	27	Report which of the following are publicly available and where they can be found: template data collection forms; data extracted from included studies; data used for all analyses; analytic code; any other materials used in the review.	Not Applicable

From: Page MJ, McKenzie JE, Bossuyt PM, Boutron I, Hoffmann TC, Mulrow CD, et al. The PRISMA 2020 statement: an updated guideline for reporting systematic reviews. BMJ 2021;372:n71. doi: 10.1136/bmj.n71

Table S2. Full search strategy

Date	Database	Search term	Results
December 15, 2022	PubMed	("vaccines"[MeSH Terms] OR "vaccin*"[Title/Abstract] OR "immunis*"[Title/Abstract] OR "immuniz*"[Title/Abstract] OR "inoculat*"[Title/Abstract]) AND ("economic evaluation"[Title/Abstract] OR "cost-utility analysis"[Title/Abstract] OR "cost-effectiveness analysis"[Title/Abstract] OR "cost-benefit analysis"[Title/Abstract] OR "cost-utility"[Title/Abstract] OR "cost-effectiveness"[Title/Abstract] OR "cost-benefit"[Title/Abstract] OR "extended cost-effectiveness analysis"[Title/Abstract] OR "distributional cost-effectiveness analysis"[Title/Abstract]) AND ("equit*"[All Fields] OR ("inequitable"[All Fields] OR "inequitably"[All Fields]) OR "distribution*"[All Fields] OR ("inequalities"[All Fields] OR "inequality"[All Fields] OR "inequities"[All Fields] OR "inequity"[All Fields]) OR "unequal distribution"[All Fields] OR ("unequal"[All Fields] OR "unequally"[All Fields] OR "unequals"[All Fields]))	375
December 15, 2022	Embase	(vaccin*:ti,ab OR immunis*:ti,ab OR immuniz*:ti,ab OR inoculat*:ti,ab) AND (‘economic evaluation’:ti,ab OR ‘cost-utility analysis’:ti,ab OR ‘cost-effectiveness analysis’:ti,ab OR ‘cost-benefit analysis’:ti,ab OR cost-utility:ti,ab OR cost-effectiveness:ti,ab OR cost-benefit:ti,ab OR ‘extended cost-effectiveness analysis’:ti,ab OR ’distributional cost-effectiveness analysis’:ti,ab) AND (equit* OR inequitable OR distribution* OR inequality OR "unequal distribution" OR unequal) AND [embase]/lim NOT ([embase]/lim AND [medline]/lim)	143
December 15, 2022	EconLit	(TI (vaccin* OR immunis* OR immuniz* OR inoculat*) OR AB (vaccin* OR immunis* OR immuniz* OR inoculat*)) AND (TI ("economic evaluation" OR "cost-utility analysis" OR "cost-effectiveness analysis" OR "cost-benefit analysis" OR cost-utility OR cost-effectiveness OR cost-benefit OR "extended cost-effectiveness analysis" OR "distributional cost-effectiveness analysis") OR ("economic evaluation" OR "cost-utility analysis" OR "cost-effectiveness analysis" OR "cost-benefit analysis" OR cost-utility OR cost-effectiveness OR cost-benefit OR "extended cost-effectiveness analysis" OR "distributional cost-effectiveness analysis")) AND (TX equit* OR inequitable OR distribution* OR inequality OR "unequal distribution" OR unequal)	39
December 15, 2022	Cost-Effectiveness Analysis Registry by Tufts Medical Center	Intervention Type is: Immunization and Abstract is: Equitable	3
		Intervention Type is: Immunization and Abstract is: Equity	4
		Intervention Type is: Immunization and Abstract is: Inequitable	1
		Intervention Type is: Immunization and Abstract is: Distribution	27
		Intervention Type is: Immunization and Abstract is: Extended	20
		Intervention Type is: Immunization and Abstract is: Inequality	1
		Intervention Type is: Immunization and Abstract is: Unequal	0
		**TOTAL**	613

Table S3. Excluded studies with reasons

Reason for exclusion	Citation
No health equity impact (n = 29)	1. Kim SY, Sweet S, Chang J, Goldie SJ. Comparative evaluation of the potential impact of rotavirus versus HPV vaccination in GAVI-eligible countries: a preliminary analysis focused on the relative disease burden. BMC Infect Dis. 2011;11:174.
	2. Jit M, Brisson M, Portnoy A, Hutubessy R. Cost-effectiveness of female human papillomavirus vaccination in 179 countries: a PRIME modelling study. Lancet Glob Health. 2014;2(7):e406-14.
	3. Datta S, Pink J, Medley GF, Petrou S, Staniszewska S, Underwood M, et al. Assessing the cost-effectiveness of HPV vaccination strategies for adolescent girls and boys in the UK. BMC Infect Dis. 2019;19(1):552.
	4. Debellut F, Clark A, Pecenka C, Tate J, Baral R, Sanderson C, et al. Re-evaluating the potential impact and cost-effectiveness of rotavirus vaccination in 73 Gavi countries: a modelling study. Lancet Glob Health. 2019;7(12):e1664-e74.
	5. Espana G, Yao Y, Anderson KB, Fitzpatrick MC, Smith DL, Morrison AC, et al. Model-based assessment of public health impact and cost-effectiveness of dengue vaccination following screening for prior exposure. PLoS Negl Trop Dis. 2019;13(7):e0007482.
	6. Gouveia M, Jesus G, Ines M, Costa J, Borges M. Cost-effectiveness of the 13-valent pneumococcal conjugate vaccine in adults in Portugal versus "no vaccination" and versus vaccination with the 23-valent pneumococcal polysaccharide vaccine. Hum Vaccin Immunother. 2019;15(4):850-8.
	7. Hoshi SL, Seposo X, Shono A, Okubo I, Kondo M. Cost-effectiveness of Recombinant Zoster Vaccine (RZV) and Varicella Vaccine Live (VVL) against herpes zoster and post-herpetic neuralgia among adults aged 65 and over in Japan. Vaccine. 2019;37(27):3588-97.
	8. Ansaldi F, Pugh S, Amicizia D, Di Virgilio R, Trucchi C, Orsi A, et al. Estimating the Clinical and Economic Impact of Switching from the 13-Valent Pneumococcal Conjugate Vaccine (PCV13) to the 10-Valent Pneumococcal Conjugate Vaccine (PCV10) in Italy. Pathogens. 2020;9(2).
	9. Beresniak A, Rizzo C, Oxford J, Gorynski P, Pistol A, Fabiani M, et al. Cost-effectiveness of public health interventions against human influenza pandemics in France: a methodological contribution from the FLURESP European Commission project. Eur J Public Health. 2020;30(1):43-9.
	10. Bulula N, Mwiru DP, Swalehe O, Thomas Mori A. Vaccine storage and distribution between expanded program on immunization and medical store department in Tanzania: a cost-minimization analysis. Vaccine. 2020;38(51):8130-5.
	11. Crepey P, Redondo E, Diez-Domingo J, Ortiz de Lejarazu R, Martinon-Torres F, Gil de Miguel A, et al. From trivalent to quadrivalent influenza vaccines: Public health and economic burden for different immunization strategies in Spain. PLoS One. 2020;15(5):e0233526.

Reason for exclusion	Citation
	12. Garcia Farinas A, Linares-Perez N, Clark A, Toledo-Romani ME, Omeiri NE, Marrero Araujo MC, et al. Cost-effectiveness of introducing a domestic pneumococcal conjugate vaccine (PCV7-TT) into the Cuban national immunization programme. Int J Infect Dis. 2020;97:182-9.
	13. Lu CY, Chung CH, Huang LM, Kruger E, Tan SC, Zhang XH, et al. Cost-effectiveness evaluation of the 10-valent pneumococcal non-typeable Haemophilus influenzae protein D conjugate vaccine for children in Taiwan. Cost Eff Resour Alloc. 2020;18:30.
	14. Pugh S, Wasserman M, Moffatt M, Marques S, Reyes JM, Prieto VA, et al. Estimating the Impact of Switching from a Lower to Higher Valent Pneumococcal Conjugate Vaccine in Colombia, Finland, and The Netherlands: A Cost-Effectiveness Analysis. Infect Dis Ther. 2020;9(2):305-24.
	15. Shami JJP, Pathadka S, Chan EW, Hui J, Sato R, Patil S, et al. Evaluating the cost-effectiveness of a sequential pneumococcal vaccination compared to single-dose vaccination strategy for adults in Hong Kong. Hum Vaccin Immunother. 2020;16(8):1937-44.
	16. Amiche A, Tanriover MD, Bellier L, Ugur B, Akin L. Cost Utility of Switching From Trivalent to Quadrivalent Influenza Vaccine in Turkey. Value Health Reg Issues. 2021;25:15-22.
	17. Kim SY, Min KD, Jung SM, Russell LB, Toscano C, Minamisava R, et al. Cost-effectiveness of maternal pertussis immunization: Implications of a dynamic transmission model for low- and middle-income countries. Vaccine. 2021;39(1):147-57.
	18. Kohli MA, Maschio M, Mould-Quevedo JF, Ashraf M, Drummond MF, Weinstein MC. The Cost-Effectiveness of Expanding Vaccination with a Cell-Based Influenza Vaccine to Low Risk Adults Aged 50 to 64 Years in the United Kingdom. Vaccines (Basel). 2021;9(6).
	19. Langsam D, Kahana D, Shmueli E, Yamin D. Cost-Effectiveness of Pertussis Vaccination Schedule in Israel. Vaccines (Basel). 2021;9(6).
	20. Luyten J, van Hoek AJ. Integrating Alternative Social Value Judgments Into Cost-Effectiveness Analysis of Vaccines: An Application to Varicella-Zoster Virus Vaccination. Value Health. 2021;24(1):41-9.
	21. Pearson CAB, Bozzani F, Procter SR, Davies NG, Huda M, Jensen HT, et al. COVID-19 vaccination in Sindh Province, Pakistan: A modelling study of health impact and cost-effectiveness. PLoS Med. 2021;18(10):e1003815.
	22. Reddy KP, Fitzmaurice KP, Scott JA, Harling G, Lessells RJ, Panella C, et al. Clinical outcomes and cost-effectiveness of COVID-19 vaccination in South Africa. Nat Commun. 2021;12(1):6238.
	23. Ryckman T, Karthikeyan AS, Kumar D, Cao Y, Kang G, Goldhaber-Fiebert JD, et al. Comparison of Strategies for Typhoid Conjugate Vaccine Introduction in India: A Cost-Effectiveness Modeling Study. J Infect Dis. 2021;224(Supple 5):S612-S24.

Reason for exclusion	Citation
	24. Daniels V, Saxena K, Patterson-Lomba O, Gomez-Lievano A, Saah A, Luxembourg A, et al. Modeling the health and economic implications of adopting a 1-dose 9-valent human papillomavirus vaccination regimen in a high-income country setting: An analysis in the United Kingdom. Vaccine. 2022;40(14):2173-83.
	25. Du Z, Wang L, Pandey A, Lim WW, Chinazzi M, Piontti APY, et al. Modeling comparative cost-effectiveness of SARS-CoV-2 vaccine dose fractionation in India. Nat Med. 2022;28(5):934-8.
	26. Hoshi SL, Shono A, Seposo X, Okubo R, Kondo M. Cost-effectiveness analyses of 15- and 20-valent pneumococcal conjugate vaccines for Japanese elderly. Vaccine. 2022;40(49):7057-64.
	27. Linertova R, Guirado-Fuentes C, Mar-Medina J, Teljeur C. Cost-effectiveness and epidemiological impact of gender-neutral HPV vaccination in Spain. Hum Vaccin Immunother. 2022;18(6):2127983.
	28. Sargazi N, Takian A, Daroudi R, Nahvijou A, Yaseri M, Ghanbari Motlagh A, et al. Cost-Benefit Analysis of Human Papillomavirus Vaccine in Iran. J Prev (2022). 2022;43(6):841-57.
	29. Siedner MJ, Alba C, Fitzmaurice KP, Gilbert RF, Scott JA, Shebl FM, et al. Cost-effectiveness of Coronavirus Disease 2019 Vaccination in Low- and Middle-Income Countries. J Infect Dis. 2022;226(11):1887-96.

Table S4. Characteristics of the included studies

First author, Year, Country	Comparisons of vaccination programs	Equity-relevant groups	Existing inequities	Perspective of analysis†	Health benefits	Non-health benefits	Model type	Inclusion of herd protection
**Health equity impact analysis**								
Anderson, 2020[20], Nigeria	Base-case analysis<br>- Introduction of different rotavirus vaccines<br>- No vaccination<br>Scenario analysis<br>- Improving vaccination coverage	Regions, Income quintiles	Mortality, Vaccination coverage	Health system	Deaths averted, DALYs averted	None	Static	No
Bell, 2020[21], Malawi	- 10 combination of malaria vaccination strategies (no. of doses and interval) and bed net usage<br>- No vaccination and no bed net	Rural/Urban	Incidence	Health system	Cases averted	None	Static	No
Blakely, 2014[22], New Zealand	- Current HPV vaccination program<br>- School-based only with equitable coverage<br>- Mandatory school-based with permitted opt-out and equitable coverage<br>- No vaccination	Māori/non-Māori, Income tertiles	Incidence, Mortality, Vaccination coverage	Health system	QALYs gained	None	Static	Yes (base-case analysis)
Goldie, 2011[23], United States	- 3 Combinations of HPV vaccination with screening patterns<br>- No vaccination with new screening algorithm and targeted risk-based protocols<br>- No vaccination with current screening patterns	Black/White/Hispanic	Incidence, Mortality, Screening coverage	Health system	Cases averted, Years of life saved	None	Static	No
Rheingans, 2012[24], 25 low- and middle-income countries	- Current rotavirus vaccination program<br>- No vaccination<br>- Equalized vaccination coverage to the highest quintile's coverage	Income quintile (25 countries), States (India)	Mortality, Vaccination coverage	Health system	Deaths averted, DALYs averted	None	Static	No
Rheingans, 2014[25], India	- Introduction of rotavirus vaccination<br>- Eliminating geographic and socioeconomic disparities across subpopulations	Rural/Urban, Regions, Gender,	Mortality, Vaccination coverage	Health system	Deaths averted, DALYs averted	None	Static	No

First author, Year, Country	Comparisons of vaccination programs	Equity-relevant groups	Existing inequities	Perspective of analysis†	Health benefits	Non-health benefits	Model type	Inclusion of herd protection
	- No vaccination	Income quintiles						
Rheingans, 2018[26], Lao PDR	- Introduction of rotavirus vaccination<br>- Improving vaccination coverage in those who are not vaccinated (under-coverage) by 10% increments<br>- No vaccination	Rural/Urban, Income quintiles	Mortality, Vaccination coverage	Health system	Deaths averted, DALYs averted	None	Static	No
Rheingans, 2018[27], Pakistan	- Introduction of rotavirus vaccination<br>- Equitable vaccination coverage<br>- No vaccination	Rural/Urban, Income quintiles	Mortality	Health system	Deaths averted, DALYs averted	None	Static	No
Urueña, 2015[28], Argentina	- Introduction of rotavirus vaccines<br>- No vaccination	Region	Incidence, Mortality	Societal	Hospitalizations and outpatient/clinic visits averted, Deaths averted, DALYs averted	None	Static	Yes (scenario analysis)
Wateska, 2019[29], United States	- PCV13 and PPSV23 for immunocompromised persons / PPSV23 for other high-risk conditions<br>- PPSV for immunocompromised persons and other high-risk conditions<br>- PCV13 and PPSV23 for immunocompromised persons and other high-risk conditions<br>- PPSV23 for everyone at age 50 - PCV13 and PPSV23 for everyone at age 50<br>- No vaccination	Black vs General population	Incidence, Mortality	Health system	Cases averted, Deaths averted, QALYs gained	None	Static	No
Wateska, 2022[30], United States	- PCV13 and PPSV23 for immunocompromised persons / PPSV23 for other high-risk conditions<br>- PPSV for immunocompromised persons and other high-risk conditions<br>- PCV13 and PPSV23 for immunocompromised persons and other high-risk conditions	Black vs non-Black	Incidence, Mortality	Health system	Cases averted, Deaths averted, QALYs gained	None	Static	Yes (scenario analysis)

First author, Year, Country	Comparisons of vaccination programs	Equity-relevant groups	Existing inequities	Perspective of analysis†	Health benefits	Non-health benefits	Model type	Inclusion of herd protection
	- PPSV23 for everyone at age 50<br>- PCV13 and PPSV23 for everyone at age 50<br>- Status quo (prior recommendation (PPSV23 to 50-year-old adults with high risk and PCV13 and PPSV23 for immunocompromised adults)							
**Health equity impact analysis with financial risk protection (Extended cost-effectiveness analysis, ECEA)**								
Assebe, 2020[3], Ethiopia	- Introduction of malaria interventions (artemisinin-based combination therapy, long-lasting insecticide-treated bed nets, indoor residual spraying, hypothetical malaria vaccine)<br>- No intervention	Income quintiles	Prevalence, Financial risk	Societal	Deaths averted	Household OOP expenditures averted, Catastrophic health expenditures averted	Static	No
Chang, 2017[31], 41 low- and middle-income countries	- Introduction of 10 vaccines for measles, hepatitis B, HPV, yellow fever, Hemophilus influenzae type b, Streptococcus pneumoniae, rotavirus, rubella, Neisseria meningitidis serogroup A, and Japanese encephalitis.<br>- No vaccination	Income quintiles	Incidence, Mortality, Vaccination coverage, Financial risk	Societal	Deaths averted	Impoverishments averted	Static	No
Driessen, 2015[4], Ethiopia	- Routine immunization with financial incentives<br>- Mass campaigns, known as supplemental immunization activities (SIAs).<br>- Routine immunization	Income quintiles	Prevalence, Mortality, Vaccination coverage, Financial risk	Societal	Deaths averted	Household OOP expenditures averted, Household OOP expenditures averted as a percentage of household income	Static	No
Johasson, 2015[5], Ethiopia	- Introduction of PCV<br>- No vaccination	Income quintiles	Mortality, Vaccination coverage,	Societal	Deaths averted	Household OOP expenditures averted, Money-metric value of insurance	Static	No
Levin, 2015[6], China	- Introduction of HPV vaccine<br>- No vaccination	Income quintiles	Mortality, Financial risk	Societal	Deaths averted	Household OOP expenditures averted, Household OOP expenditures averted as a percentage of household income	Static	No

First author, Year, Country	Comparisons of vaccination programs	Equity-relevant groups	Existing inequities	Perspective of analysis[†]	Health benefits	Non-health benefits	Model type	Inclusion of herd protection
Loganathan, 2016[7], Malaysia	- Universal public finance of rotavirus vaccine<br>- No vaccination	Income quintiles	Financial risk	Household	Hospitalizations and outpatient/clinic visits averted	Household OOP expenditures averted, Catastrophic health expenditures averted, Impoverishments averted	Static	Yes (scenario analysis)
Pecenka, 2015[8], Ethiopia	- Universal public finance of rotavirus vaccine with diarrhea treatment<br>- Diarrhea treatment without no vaccination	Income quintiles	Mortality, Vaccination coverage, Financial risk	Societal	Deaths averted	Household OOP expenditures averted	Static	No
Portnoy, 2021[9], Ethiopia	- Routine immunization of HPV vaccine<br>- Currently implemented vaccination campaign	Income quintiles	Prevalence, Vaccination coverage, Financial risk	Societal	Cases averted	Household OOP expenditures averted, Catastrophic health expenditures averted	Static	No
Verguet, 2013[10], India and Ethiopia	- Public finance of rotavirus vaccine<br>- No vaccination	Income quintiles	Mortality, Financial risk	Societal	Deaths averted	Household OOP expenditures averted, Money-metric value of insurance	Static	Yes (scenario analysis)
**Health equity impact analysis with equity-weighting (Distributional cost-effectiveness analysis, DCEA)**								
Dawkins, 2018[32], Ethiopia	- Pro-poor vaccination program of rotavirus<br>- Currently implemented rotavirus vaccination program	Income quintiles	Incidence, Mortality, Vaccination coverage	Health system	Deaths averted, HALYs averted	None	Static	No

[†]Perspective was categorized based on authors' statements in the articles or reviewers' judgment based on methodologies of the studies. Abbreviations: DALY – disability-adjusted life year; HALY – health-adjusted life year; HPV – human papillomavirus; OOP – out-of-pocket; PCV – pneumococcal conjugate vaccine; PPSV – pneumococcal polysaccharide vaccine; QALY- quality-adjusted life year.

Table S5. Summary of antigen by income economy

Antigen*	High income countries (n = 4)	Low- and middle income countries (n = 17)	Total (N = 21)
Rotavirus	-	11 (65%)	11 (52%)
Human papilloma virus	2 (50%)	3 (18%)	5 (24%)
Streptococcus pneumoniae	2 (50%)	2 (12%)	4 (19%)
Malaria	-	2 (12%)	2 (10%)
Measles	-	2 (12%)	2 (10%)
Hepatitis B	-	1 (6%)	1 (5%)
Hemophilus influenzae type b	-	1 (6%)	1 (5%)
Yellow fever	-	1 (6%)	1 (5%)
Rubella	-	1 (6%)	1 (5%)
Neisseria meningitidis serogroup A	-	1 (6%)	1 (5%)
Japanese encephalitis	-	1 (6%)	1 (5%)

Note: *Number of studies may not add up, as some included multiple vaccines.

Table S6. Summary of findings of included studies

First author, Year, Country	Comparisons of vaccination programs	Equity-relevant groups	Existing inequities	Cost-effectiveness findings across subpopulations*	Equity impact findings across subpopulations*
**Health equity impact analysis**					
Anderson, 2020[20], Nigeria	Base-case analysis<br>- Introduction of different rotavirus vaccines<br>- No vaccination<br>Scenario analysis<br>- Improving vaccination coverage	Regions, Income quintiles	Mortality, Vaccination coverage	Base-case analysis<br>- All regional ICERs had an inverse relationship to burden, where lower ICERs (thus more favorable) were found in higher rotavirus mortality burden regions.<br>- For North Central, North West, and South South regions, ICERS were higher for children from the poorer, middle, or richer quintiles than children from the richest quintile.<br>- Due to high, persistent, and inequitable burden of rotavirus in Nigeria, routine vaccination with any of these rotavirus vaccines would be an high impact and cost-effective strategy in reducing child mortality.	Base-case analysis<br>- Within regions, the highest benefit was found in the poorest and poorer quintiles in South South and South West and in the richest quintile in North West.<br>- Mortality reductions were highest for children in the richest quintiles in the three southern regions<br>- Disparities in mortality reduction were largely driven by inequality in vaccination coverage across regions and between socioeconomic subpopulations.<br><br>Scenario analysis<br>- Improvements in coverage had the largest effects in the North East and North West regions, where estimated rotavirus vaccine coverage was the lowest.<br>- A 50% improvement in coverage would result in deaths averted per 1000 rates that are 3.5 and 2.5 times higher in North West and North East, respectively.
Bell, 2020[21], Malawi	- 10 combination of malaria vaccination strategies (no. of doses and interval) and bed net usage<br>- No vaccination and no bed net	Rural/Urban	Incidence	Since malaria incidence in rural Lilongwe is higher than in urban Lilongwe, the impact and cost-effectiveness of vaccine interventions is increased in rural areas.	Since malaria incidence in rural Lilongwe is higher than in urban Lilongwe, the impact and cost-effectiveness of vaccine interventions is increased in rural areas.
Blakely, 2014[22], New Zealand	- Current HPV vaccination program<br>- School-based only with equitable coverage	Māori/non-Māori, Income tertiles	Incidence, Mortality, Vaccination coverage	The ICERs do not vary greatly by ethnicity or deprivation–although the ICERs for Māori are lower than for the total population.	Regarding differences by ethnicity and deprivation, all three interventions appear pro-equity in that there were greater health gains for Māori and the living in the most deprived areas (tertile 3) compared to no HPV vaccination program.

First author, Year, Country	Comparisons of vaccination programs	Equity-relevant groups	Existing inequities	Cost-effectiveness findings across subpopulations*	Equity impact findings across subpopulations*
	- Mandatory school-based with permitted opt-out and equitable coverage<br>- No vaccination				
Goldie, 2011[23], United States	- 3 Combinations of HPV vaccination with screening patterns<br>- No vaccination with new screening algorithm and targeted risk-based protocols<br>- No vaccination with current screening patterns	Black/White/ Hispanic	Incidence, Mortality, Screening coverage	Subpopulation ICERs were not quantified.	With respect to the distribution of outcomes across subgroups, disparities were widest for Hispanic women (increase from 54.8% to 63.9% in cancer incidence reduction), followed by black women (increase from 60.1% to 68.3%), compared to white women (increase from 62.5% to 71.6% in cancer incidence reduction).
Rheingans, 2012[24], 25 low- and middle-income countries	- Current rotavirus vaccination program<br>- No vaccination<br>- Equalized vaccination coverage to the highest quintile's coverage	Income quintile (25 countries), States (India)	Mortality, Vaccination coverage	<u>Current vs no vaccination</u><br>- In most countries, the CER is highest (least cost-effective) for the richest quintile and the benefit is the lowest, primarily due to lower estimated mortality rates.<br>- Cost-effectiveness and benefits differed substantially among states, from over $250/DALY averted in Kerala to less than $60/DALY averted in Madhya Pradesh. The states with the lowest CERs are those with high pre-vaccination mortality (larger circles).	<u>Current vs no vaccination</u><br>- In poorer quintiles, the benefit tends to go up due to increased mortality, but sometimes goes down due to lower vaccination coverage rates.<br>- However, many of these same states also have the lowest percent reduction in rotavirus mortality, due to low vaccination coverage. If national rotavirus vaccination were implemented on top of existing EPI coverage, then the states with the most favorable cost-effectiveness ratios and greatest burden would actually benefit the least.<br><br><u>Equalized vaccination coverage vs no vaccination</u><br>- In Chad, Nigeria, DRC, India and Niger is substantial, where equitable coverage could improve mortality reduction among the poorest quintile by 656%, 460%, 96%, 90% and 89%, respectively. In contrast, the potential increase in impact in the poorest

First author, Year, Country	Comparisons of vaccination programs	Equity-relevant groups	Existing inequities	Cost-effectiveness findings across subpopulations*	Equity impact findings across subpopulations*
					quintile, due to more equitable vaccine coverage, was less than 5% in Bangladesh, Uganda, and Ghana.<br>- Eliminating differences in coverage between richest and poorest quintiles could increase the number of deaths averted by 89% among the poorest quintile and could increase the overall number of lives saved by 38%.
Rheingans, 2014[25], India	- Introduction of rotavirus vaccination<br>- Eliminating geographic and socioeconomic disparities in mortality reduction across subpopulations<br>- No vaccination	Rural/Urban, Regions, Gender, Income quintiles	Mortality, Vaccination coverage	<u>Introduction vs No vaccination</u><br>- Cost effectiveness also varied within geographic areas as higher wealth quintiles typically had lower incremental costs (due to greater medical costs), yet lower health benefits (due to lower mortality).<br>- All ratios at the regional and state levels are substantially lower than the GDP per capita of $1490 in India.	<u>Introduction vs No vaccination</u><br>- We estimate that vaccine introduction will reduce rotavirus disease burden by 30% to 39% depending on the region, with the greatest percent reduction estimated in the South (39%), followed by the North (34%) and West regions (34%).<br>- The absolute level of benefits (deaths averted per 1000 births) also varied across regions, ranging from 0.55 to 1.66 rotavirus deaths per 1000 births, with the highest benefits estimated in Central, Northeast, and East regions.<br>- For all regions, the highest percent reduction in burden was estimated for the two highest wealth quintiles. The highest and most equitable reduction was estimated in the South, ranging from 38% to 40% across quintiles.<br>- The greatest potential health benefits of vaccination will come from reaching high rotavirus mortality areas and the poorest households. However, these populations are less likely to benefit given current low coverage estimates.

First author, Year, Country	Comparisons of vaccination programs	Equity-relevant groups	Existing inequities	Cost-effectiveness findings across subpopulations*	Equity impact findings across subpopulations*
					Equalized vaccination coverage vs no vaccination<br>- The highest potential additional benefits are among the high mortality regions and states, and particularly among the poorest quintiles. Nationally, increased coverage would increase benefit estimates by 23%, preventing 9400 additional deaths. In Bihar, Madhya Pradesh and Uttar Pradesh benefit estimates would increase by 55%, 76% and 71%, respectively, preventing 10,600 additional deaths.
Rheingans, 2018[26], Lao PDR	- Introduction of rotavirus vaccination<br>- Improving vaccination coverage in those who are not vaccinated (under-coverage) by 10% increments<br>- No vaccination	Rural/Urban, Income quintiles	Mortality, Vaccination coverage	Introduction vs No vaccination<br>- The incremental cost-effectiveness ratio ($/DALY) is lowest (most cost-effective) for the Central region ($124/DALY) compared to the North region ($158/DALY).<br>- The ICER varies within region and is lowest (most cost-effective) in the poorer and poorest quintiles in all regions due to the higher burden of disease.<br>- In the Central region, ICERs ranged from $78/DALY (poorest) to $144/DALY (richer), compared to the North region where ICERs range from $98/DALY to $353/DALY.<br><br>Improving vaccination coverage vs No vaccination<br>- Universal coverage would have the greatest effects on impact in the South region where full coverage resulted in 1.7 times more deaths averted, with the most improvement in cost-effectiveness in the Central region.	Introduction vs No vaccination<br>- Regionally, rotavirus vaccination will reduce disease burden by 24% (Central), 26% (North) and 32% (South), with the greatest estimated reductions in children living in the richer and richest households but the greatest benefit is in the poorer and poorest quintiles of all regions.<br><br>Improving vaccination coverage vs No vaccination<br>- Universal coverage would have the greatest effects on impact in the South region where full coverage resulted in 1.7 times more deaths averted, with the most improvement in cost-effectiveness in the Central region.

First author, Year, Country	Comparisons of vaccination programs	Equity-relevant groups	Existing inequities	Cost-effectiveness findings across subpopulations*	Equity impact findings across subpopulations*
Rheingans, 2018[27], Pakistan	- Introduction of rotavirus vaccination<br>- Equitable vaccination coverage<br>- No vaccination	Regions, Income quintiles	Mortality	<u>Introduction vs No vaccination</u><br>- Sindh and Balochistan had the lowest Gavi-perspective ICERs (most cost-effective): $155 and $167/DALY, respectively, compared to $594/DALY in Islamabad.<br>- The ICER varied within region and was lowest (most cost-effective) in the poorest quintiles in all regions due to higher disease burden.<br><br><u>Equitable vaccination coverage vs No vaccination</u><br>- This scenario assuming equal coverage reduced estimated national ICERS from $279/DALY to $203/DALY.	<u>Introduction vs No vaccination</u><br>- The greatest absolute benefits of rotavirus vaccination were in Punjab, Sindh, and Khyber Pakhtunkhwa and were generally greater for the poorest quintile (except in Khyber Pakhtunkhwa), primarily because of higher risk in these subpopulations.<br>- In contrast, percent mortality reduction was greatest in the higher wealth quintiles, where estimated coverage was the highest.<br><br><u>Equitable vaccination coverage vs No vaccination</u><br>- Full, equitable coverage would have the greatest effect in the most vulnerable regions with a 192% increase in deaths averted in Sindh and a 295% increase in Baluchistan.<br>- Within these regions, the greatest improvement would be among children in the poorest households.
Urueña, 2015[28], Argentina	- Introduction of rotavirus vaccines<br>- No vaccination	Region	Incidence, Mortality	- ICERs were lower in the North East area and North West area for both vaccines.	- Although the burden of disease is higher in the North West area than in the North East area, both vaccines would avert more DALYs in the North East area, due to higher mortality in this region.
Wateska, 2019[29], United States	- PCV13 and PPSV23 for immunocompromised persons / PPSV23 for other high-risk conditions<br>- PPSV for immunocompromised persons and other high-risk conditions	Black vs General population	Incidence, Mortality	- From a public health perspective, giving both PPSV23 and PCV13 to all 50-year-olds resulted in the fewest IPD and NBP cases and deaths for both the general and black populations and in scenarios when PPSV23 was or was not effective against NBP.	- Morbidity and mortality reductions when PPSV was effective against NBP were 1108 cases (NNV 114) and 32 deaths in the black population and 5981 cases (NNV = 156) and 189 deaths in the general population.

First author, Year, Country	Comparisons of vaccination programs	Equity-relevant groups	Existing inequities	Cost-effectiveness findings across subpopulations*	Equity impact findings across subpopulations*
	- PCV13 and PPSV23 for immunocompromised persons and other high-risk conditions<br>- PPSV23 for everyone at age 50<br>- PCV13 and PPSV23 for everyone at age 50<br>- No vaccination				
Wateska, 2022[30], United States	- PCV13 and PPSV23 for immunocompromised persons / PPSV23 for other high-risk conditions<br>- PPSV for immunocompromised persons and other high-risk conditions<br>- PCV13 and PPSV23 for immunocompromised persons and other high-risk conditions<br>- PPSV23 for everyone at age 50<br>- PCV13 and PPSV23 for everyone at age 50<br>- Status quo (prior recommendation (PPSV23 to 50-year-old adults with high risk and PCV13 and PPSV23 for immunocompromised adults)	Black vs non-Black	Incidence, Mortality	- In the Black cohort, PCV15/PPSV23 given only at age 50 years cost $104,723 per quality adjusted life (QALY) gained, while PCV15/ PPSV23 given at both ages 50/65 cost $240,952/QALY compared to PCV15/PPSV23 only at age 50.<br>- In non-Blacks, giving PCV15 and PPSV23 at ages 50/65 cost $306,017/QALY while giving the PCV15/PPSV23 combination only at age 50 cost $195,985/QALY gained.<br>- Giving PCV20 at ages 50/65 was unfavorable in both Black and non-Black populations under base case vaccine effectiveness assumptions, due to higher incremental cost-effectiveness ratios than more effective strategies (i.e., extended dominance).	- Compared to PCV15/PPSV23 at 50/65, use of either current CDC recommended strategy resulted in 1.2 % more pneumococcal disease cases and 2.2–2.4 % more pneumococcal disease deaths in the Black cohort. In the non-Black cohort, 1.0– 1.1 % more cases and 1.6–1.7 % more deaths occurred with current recommendations.
**Health equity impact analysis with financial risk protection (Extended cost-effectiveness analysis, ECEA)**					
Assebe, 2020[3], Ethiopia	- Introduction of malaria interventions (artemisinin-based combination therapy, long-lasting insecticide-treated bed nets,	Income quintiles	Prevalence, Financial risk	Subpopulation ICERs were not quantified.	- All four interventions would save larger numbers of lives among the poor, due to the fact that the poor would face a higher malaria prevalence and associated risk factors.

First author, Year, Country	Comparisons of vaccination programs	Equity-relevant groups	Existing inequities	Cost-effectiveness findings across subpopulations*	Equity impact findings across subpopulations*
	indoor residual spraying, hypothetical malaria vaccine)<br>- No intervention				- The distribution of deaths averted by the malaria vaccine would be 30, 22, 21, 16, and 11%, respectively.<br>- The poorest income quintiles would see the greatest FRP benefits.
Chang, 2017[31], 41 low- and middle-income countries	- Introduction of 10 vaccines for measles, hepatitis B, HPV, yellow fever, Hemophilus influenzae type b, Streptococcus pneumoniae, rotavirus, rubella, Neisseria meningitidis serogroup A, and Japanese encephalitis<br>- No vaccination	Income quintiles	Incidence, Mortality, Vaccination coverage, Financial risk	Subpopulation ICERs were not quantified.	- Although the poorest quintiles experienced the lowest vaccine coverage rates, they enjoyed the most health benefits in terms of absolute number of averted deaths: The poorest quintile ac- counted for the largest share of deaths averted by all vaccines (23–34 percent), and the poorest two quintiles accounted for over half of the deaths averted by most vaccines.<br>- For all antigens, the poorest quintile accounted for the greatest number of deaths averted per million vaccinated. In other words, the benefit of vaccination for a person in the poorest quintile was greater than that for a person in a richer quintile.<br>- The vast majority of averted impoverishment cases occurred in the poorest quintiles, and fewer than 20,000 cases were averted in the richest quintile.<br>- For many vaccines (for example, those for measles, hepatitis B, human papillomavirus, rotavirus, Neisseria meningitidis serogroup A, and Japanese encephalitis), more than 40 percent of the averted cases occurred in the poorest quintile.
Driessen, 2015[4], Ethiopia	- Routine immunization with financial incentives<br>- Mass campaigns, known as supplemental immunization activities (SIAs).	Income quintiles	Prevalence, Mortality, Vaccination coverage, Financial risk	Subpopulation ICERs were not quantified.	- SIAs achieve a greater health impact across all quintiles, while the routine immunization with financial incentives results in more modest health gains overall but did create additional demand in households in the lower two quintiles, which

First author, Year, Country	Comparisons of vaccination programs	Equity-relevant groups	Existing inequities	Cost-effectiveness findings across subpopulations*	Equity impact findings across subpopulations*
	- Routine immunization				ultimately generated dramatic welfare improvements through increased income due to the incentives.<br>- Sharp declines in the lower two income quintiles, the target group for the incentives; in these groups, deaths averted were almost three times higher under incentives as compared to routine immunization offered without incentives.<br>- When comparing routine immunization with and without financial incentives; expenditures averted are almost three times higher for the lower two quintiles under the incentive option due to a similar increase in coverage.
Johasson, 2015[5], Ethiopia	- Introduction of PCV<br>- No vaccination	Income quintiles	Mortality, Vaccination coverage,	Subpopulation ICERs were not quantified.	- Save more lives among the poorest groups due to higher disease burden in this population<br>- Wealthier people avert more private expenditures (around 60% of total private expenditures averted from UPF of pneumonia treatment would be felt in the two richest quintiles).<br>- There is a shift in gradients between private expenditures averted and FRP, where the poorest have in absolute terms the lowest private expenditures averted but benefit from the highest FRP.
Levin, 2015[6], China	- Introduction of HPV vaccine<br>- No vaccination	Income quintiles	Mortality, Financial risk	Subpopulation ICERs were not quantified.	- While the relative cancer reduction is constant across income groups, the absolute number of cervical cancer deaths averted and the financial risk protection from HPV vaccination are highest among women in the lowest quintile; women in the bottom income quintiles received relatively higher cost benefits compared to the upper wealth quintiles.

**First author, Year, Country**	**Comparisons of vaccination programs**	**Equity-relevant groups**	**Existing inequities**	**Cost-effectiveness findings across subpopulations***	**Equity impact findings across subpopulations***
					- HPV vaccination averts 15 percent more detected cancer cases and 18 percent more deaths in the lowest compared to the highest quintile.<br>- Although in absolute dollars, patient savings were higher in the top income quintile compared to the lowest quintile (US$7,655,200 compared to US$1,636,270), the cost savings from HPV vaccination comprised a larger share of per capita income among women in the bottom income quintiles, ranging from 60 percent among the lowest income quintile to 30 percent among the highest quintile.
Loganathan, 2016[7], Malaysia	- Universal public finance of rotavirus vaccine<br>- No vaccination	Income quintiles	Financial risk	Subpopulation ICERs were not quantified.	- We found that rotavirus vaccination resulted in substantial reduction in rotavirus episodes and expenditure across all income groups. Annually, rotavirus vaccination resulted in savings of almost US$ 6 million to households seeking care for rotavirus episodes. These benefits were evenly distributed across income quintiles.<br>- In terms of financial risk protection, vaccination averts catastrophic expenditure among all income groups. However, poverty reduction benefits were concentrated among the poorest two quintiles.
Pecenka, 2015[8], Ethiopia	- Universal public finance of rotavirus vaccine with diarrhea treatment<br>- Diarrhea treatment without no vaccination	Income quintiles	Mortality, Vaccination coverage, Financial risk	Subpopulation ICERs were not quantified.	- In terms of deaths averted, the interventions provide greater benefits to the poor, and the scale of these benefits favors rotavirus vaccination along with diarrheal treatment over diarrheal treatment alone.<br>- Per US$1 million spent across the entire population, about five times as many deaths are averted in the lowest quintile relative to the wealthiest due to UPF of diarrheal treatment.

First author, Year, Country	Comparisons of vaccination programs	Equity-relevant groups	Existing inequities	Cost-effectiveness findings across subpopulations*	Equity impact findings across subpopulations*
					- For diarrheal treatment alone, and diarrheal treatment along with rotavirus vaccination, the wealthy tend to experience the greatest gains in private expenditures averted.
Portnoy, 2021[9], Ethiopia	- Routine immunization of HPV vaccine<br>- Currently implemented vaccination campaign	Income quintiles	Prevalence, Vaccination coverage, Financial risk	Subpopulation ICERs were not quantified.	- Assuming 50% flat coverage, 31% of these health benefits would accrue to the poorest quintile compared with 43% to the richest quintile, whereas, assuming a coverage gradient averaging 50%, 15% of the health benefits would accrue to the poorest quintile compared with 61% in the richest.<br>- Routine two-dose HPV vaccination could avert $41 200 000 ($31 400 000–42 200 000; flat coverage of 50%) to $46 600 000 ($35 600 000–47 800 000; coverage gradient of 50%) in total OOP expenditures over 2019–2118 compared to no vaccination, with the bottom two quintiles accounting for ~25% of all OOP expenditures averted in the latter.<br>- When examining the FRP benefits by wealth quintile, ~33– 50% of these FRP benefits (assuming a coverage gradient or flat coverage, respectively) would be experienced by the poorest quintile.
Verguet, 2013[10], India and Ethiopia	- Public finance of rotavirus vaccine<br>- No vaccination	Income quintiles	Mortality, Financial risk	Subpopulation ICERs were not quantified.	- In India and Ethiopia, more lives would be saved among the bottom income quintile compared to the top income quintile (29% and 27% of benefits accrue to the bottom income quintile in India and Ethiopia).<br>- In India and Ethiopia, total household expenditures averted per million infants vaccinated would be $1,800,000 and $800,000, and the bottom two income quintiles would account for about 34% and 25% of all household expenditures averted.

First author, Year, Country	Comparisons of vaccination programs	Equity-relevant groups	Existing inequities	Cost-effectiveness findings across subpopulations*	Equity impact findings across subpopulations*
					- Total FRP (for 1,000,000 households) would be about $16,000 and $8000. The largest FRP value would be felt by the bottom income quintile in India (33% of total FRP) and Ethiopia (27%).
**Health equity impact analysis with equity-weighting (Distributional cost-effectiveness analysis, DCEA)**					
Dawkins, 2018[32], Ethiopia	- Pro-poor vaccination program of rotavirus<br>- Currently implemented rotavirus vaccination program	Income quintiles	Incidence, Mortality, Vaccination coverage	Subpopulation ICERs were not quantified.	- This shows that, compared with the standard program, the pro-poor program provides greater gains to the lowest wealth quintile groups at the expense of the higher wealth quintile groups.<br>- The pro-poor vaccine falls in the south-east 'lose-win' quadrant (CET 1⁄4 $50), demonstrating that relative to the standard vaccination program it has a positive impact on health equity despite its negative impact on total health. Thus, a trade-off occurs between improving total health and reducing socioeconomic inequality in health.

Note: * - Findings were excerpted from articles. Abbreviations: CER – cost-effectiveness ratio; DALY – disability-adjusted life year; GDP – gross domestic product; HALY – health-adjusted life year; HPV – human papillomavirus; ICER – incremental cost-effectiveness ratio; NBP – nonbacteremic pneumococcal pneumonia; NNV – number needed to vaccinate; OOP – out-of-pocket; PCV – pneumococcal conjugate vaccine; PPSV – pneumococcal polysaccharide vaccine; QALY- quality-adjusted life year.

Table S7. Reporting quality assessment using CHEERS 2022 statement

Topics	Anderson, 2020[20]	Bell, 2020[21]	Blakely, 2014[22]	Goldie, 2011[23]	Rheingans, 2012[24]	Rheingans, 2014[25]	Rheingans, 2018[26]	Rheingans, 2018[27]	Urueña, 2015[28]	Wateska, 2019[29]	Wateska, 2022[30]	Assebe, 2020[3]	Chang, 2017[31]	Driessen, 2015[4]	Johasson, 2015[5]	Levin, 2015[6]	Loganathan, 2016[7]	Pecenka, 2015[8]	Portnoy, 2021[9]	Verguet, 2013[10]	Dawkins, 2018[32]
**Title**																					
**Abstract**																					
**Introduction**																					
Background and objectives																					
**Methods**																					
Health economic analysis plan																					
Study population																					
Setting and location																					
Comparators																					
Perspective																					
Time horizon																					
Discount rate																					
Selection of outcomes																					
Measurement of outcomes																					
Valuation of outcomes																					
Measurement and valuation of resources and costs																					

**Topics**	**Anderson, 2020[20]**	**Bell, 2020[21]**	**Blakely, 2014[22]**	**Goldie, 2011[23]**	**Rheingans, 2012[24]**	**Rheingans, 2014[25]**	**Rheingans, 2018[26]**	**Rheingans, 2018[27]**	**Urueña, 2015[28]**	**Wateska, 2019[29]**	**Wateska, 2022[30]**	**Assebe, 2020[3]**	**Chang, 2017[31]**	**Driessen, 2015[4]**	**Johasson, 2015[5]**	**Levin, 2015[6]**	**Loganathan, 2016[7]**	**Pecenka, 2015[8]**	**Portnoy, 2021[9]**	**Verguet, 2013[10]**	**Dawkins, 2018[32]**
Currency, price date, and conversion																					
Rationale and description of model																					
Analytics and assumptions																					
Characterising heterogeneity																					
Characterising distributional effects																					
Characterising uncertainty																					
Approach to engagement with patients and others affected by the study																					
**Results**																					
Study parameters																					
Summary of main results																					
Effect of uncertainty																					

Topics	Anderson, 2020[20]	Bell, 2020[21]	Blakely, 2014[22]	Goldie, 2011[23]	Rheingans, 2012[24]	Rheingans, 2014[25]	Rheingans, 2018[26]	Rheingans, 2018[27]	Urueña, 2015[28]	Wateska, 2019[29]	Wateska, 2022[30]	Assebe, 2020[3]	Chang, 2017[31]	Driessen, 2015[4]	Johasson, 2015[5]	Levin, 2015[6]	Loganathan, 2016[7]	Pecenka, 2015[8]	Portnoy, 2021[9]	Verguet, 2013[10]	Dawkins, 2018[32]
Effect of engagement with patients and others affected by the study																					
**Discussion**																					
Study findings, limitations, generalisability, and current knowledge																					
**Other relevant information**																					
Source of funding																					
Conflicts of interest																					

Note: Green cells indicate “Yes”; Red cells indicate “No”.
